# Bananas decrease acetaminophen potency in *in vitro* assays

**DOI:** 10.1371/journal.pone.0205612

**Published:** 2018-10-12

**Authors:** Yoshihiro Uesawa, Naotaka Tsuji

**Affiliations:** 1 Medical Molecular Informatics, Meiji Pharmaceutical University, Noshio, Kiyose, Tokyo, JAPAN; 2 Sakae Pharmacy, Honcho, Higashimurayama, Tokyo, Japan; Jadavpur University, INDIA

## Abstract

Edible portions of bananas contain high levels of polyphenol oxidase, which catalyzes reactions in the melanin formation pathway. Tyrosine, a physiological substrate of polyphenol oxidase, has an analogous structure to acetaminophen. We investigated whether banana extract causes structural changes in acetaminophen and a decrease in its potency. Acetaminophen concentration in banana extract was measured under different conditions to characterize incompatibility. Reaction products in solution were identified using liquid chromatography/electrospray ionization/mass spectrometry (LC/ESI/MS). Acetaminophen potency decreased with time in the presence of banana extract. The reaction proceeded most efficiently in temperatures 30–37°C and neutral to weakly acidic conditions. Molecular ion peaks derived from the oxidized catechol moiety of acetaminophen were identified in LC/ESI/MS spectra. Our findings suggest that incorporation or simultaneous administration of acetaminophen medication and banana juice may result in decreased efficacy of the clinically important drug. This interaction is likely due to the oxidation of acetaminophen by polyphenol oxidase activity in banana pulp. Therefore, we investigated and characterized a novel interaction between bananas and acetaminophen. To establish a safe and effective antipyretic analgesic regimen using acetaminophen, future studies of this interaction are expected to be performed in humans.

## Introduction

Acetaminophen is one of the drugs most often prescribed as an antipyretic analgesic for the treatment of common cold symptoms. In addition, it is a major component of over-the-counter (OTC) pediatric cold medications, and non-medical personnel are limited to administering it as an antipyretic at infant and pediatric dosage. Meanwhile, bananas are highly nutritious and considered useful and beneficial dietary supplements during times of illness. Furthermore, they are easily incorporated into juices and other edibles and recommended as baby food; therefore, bananas are frequently used as a medium for admixing drugs to aid their administration to infants and children. Interestingly, banana pulp contains polyphenol oxidase [1], which rapidly converts catechols with two phenolic hydroxyl groups in the ortho- position into melanin precursors. Thus, banana components have been shown to interact and attenuate the actions of catecholic medications such as levodopa and reduce drug bioavailability and efficacy [2, 3].

**Fig 1:**
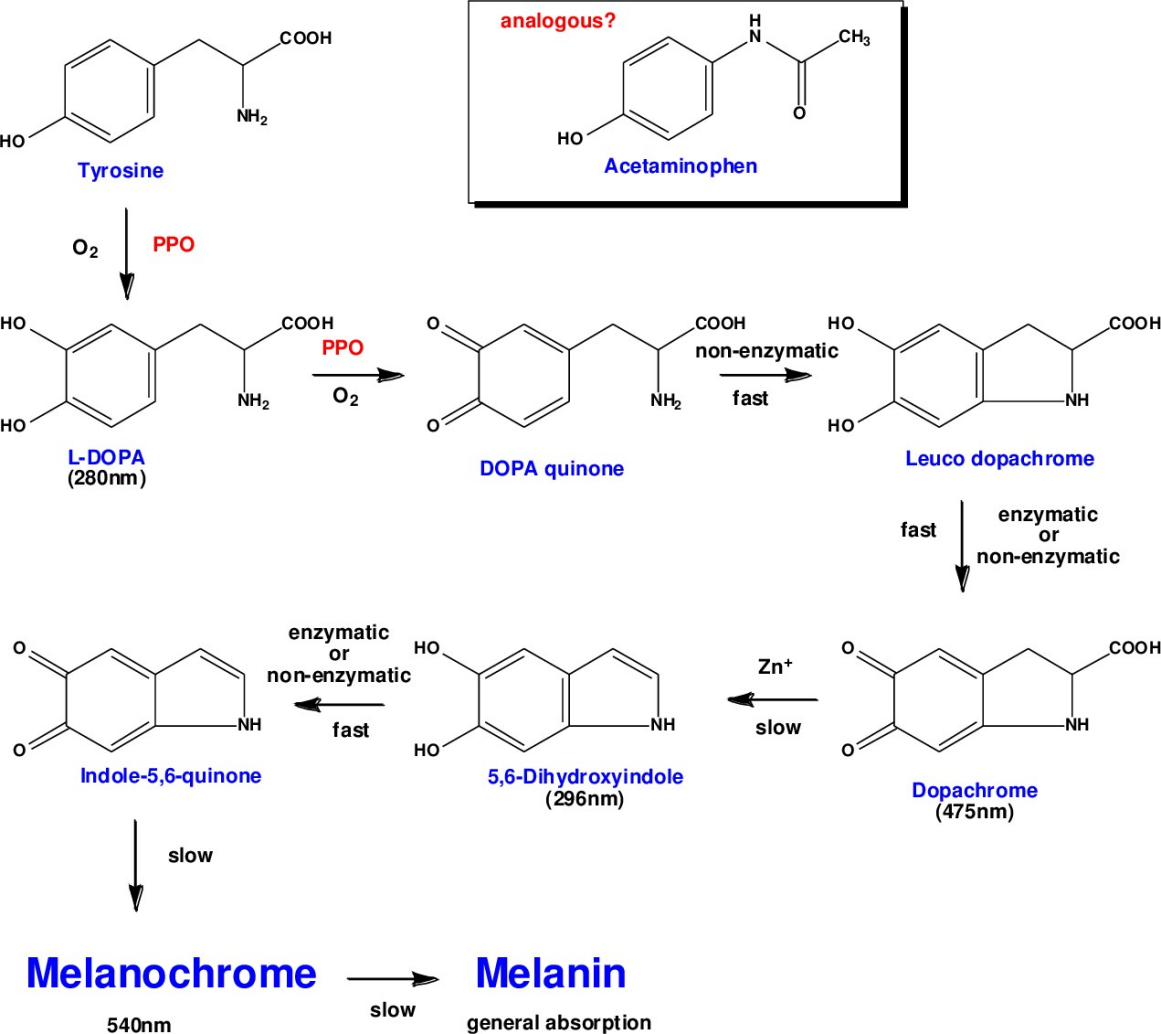
Melanin formation pathway. Acetaminophen has a chemical structure similar to that of tyrosine, a melanin precursor.

Polyphenol oxidase is the enzyme responsible for the initial stages of the melanin formation pathway; it contributes to the browning of many fruits including bananas, apples, and peaches [4]. Moreover, the phenolic amino acid tyrosine is converted to levodopa by polyphenol oxidase *in vivo* (Fig 1) [5]. Acetaminophen is chemically analogous to tyrosine in several ways: it has similar molecular size (the molecular weights of acetaminophen and tyrosine are 151.2 and 181.2 g/mol, respectively), it is a phenolic compound, and considering tautomers, hydrogen bonding donor and acceptor functional groups can be assumed to be located at positions similar to those of tyrosine. Therefore, we surmised that acetaminophen may provide a good substrate for tyrosinase in banana pulp. Indeed, a preliminary study have indicated a rapid decline in acetaminophen concentration in banana juice [6]. In this study, we attempted to elucidate the mechanism of this new incompatibility and considered its clinical impact.

## Materials and methods

### Experimental materials

Acetaminophen was obtained from MP Biomedicals, Inc. (CA, USA). 3-Hydroxyacetaminophen was purchased from Toronto Research Chemicals Inc. (Ontario, Canada). Acetonitrile of LCMS grade was used (Wako Pure Chemical Industries (Osaka, Japan). The different brands of banana fruits were purchased from 18 local markets. All other materials used were reagent grade.

### Preparation of banana supernatants

Low-viscosity 50% supernatants were prepared from centrifuged banana homogenates to allow the use of a micropipette for quantitative studies. Specifically, homogenates of edible bananas were centrifuged at 16,000 g at 4°C for 5 min and the resulting supernatants were diluted to 50% by adding equal amounts of distilled water or 50 mM Tris/MES buffer at pH 3, 4, 5, 6, 7, and 8. The prepared 50% supernatants were stored at −30°C until further use.

### Assays for acetaminophen and 3-hydroxyacetaminophen

Acetaminophen was incubated in 50% banana supernatant samples. After incubation, 10 times volume of ice-cold acetonitrile was added to the reaction mixture. The sample was mixed vigorously for 20 s and centrifuged at 16,000 g for 5 min at 4°C; then the supernatant (5 uL) was injected into LC/ESI/MS. ESI mass spectra were obtained using Shimadzu LCMS-2010EV LCMS system with an ESI probe (Shimadzu Co. Ltd.) equipped with a reversed-phase analytical Capcell Pak MGII-ODS column [2.0 mm (inside diameter)x15 cm; particle size 5 μm (Shiseido Co. Ltd., Kyoto, Japan)]. The flow rate was set at 0.2 mL/min. [M-H]+ ions at m/z 152 for acetaminophen were monitored for positive ions; the interface voltage was 4.5 kV, and the detector voltage was 1.5 kV. Negative and positive ions from 3-hydroxyacetaminophen in the reaction mixtures incubated with banana supernatant and acetaminophen were also monitored in negative and positive modes, respectively, with same conditions in scan mode. The heat block and CDL temperatures were 200 and 250°C, respectively. Nitrogen was used as the nebulization gas at flow rates of 1.5 L/min. A mobile phase consisting of water and acetonitrile was pumped through the column at a flow rate of 0.2 mL/min using a gradient from 10 to 100% acetonitrile in 8 min and subsequently 100% for 12 min.

### Effects of banana on acetaminophen potency

a) Incompatibility observed after enzyme deactivation in banana extract

- Pulp homogenate of bananas grown in the Philippines was centrifuged at 16,000 g and the resulting supernatant was used as the stock solution for reaction experiments.
- A portion of the stock solution was autoclaved at 121°C for 20 min.
- Acetaminophen was dissolved to a final concentration of 0.1 mM in 50% fresh or autoclaved banana pulp supernatants, which were then incubated at 30°C for 30 min. The residual acetaminophen was then measured. The experiment was conducted three times.

b) Effect of temperature on acetaminophen loss in banana extract

- Pulp homogenate of bananas grown in the Philippines was centrifuged at 16,000 g and the resulting supernatant was used as the stock solution for reaction experiments.
- Acetaminophen was dissolved to a final concentration of 1 mM in 50% banana pulp supernatants, which were then incubated at 0, 25, 30, 37, 50, or 60°C for 0–120 min. The residual acetaminophen was then determined. The experiment was conducted three times.

c) Effect of pH on acetaminophen loss in banana juice

- Pulp homogenate of bananas grown in the Philippines was centrifuged at 16,000 g and the resulting supernatant was adjusted to pH 3, 4, 5, 6, 7, or 8 using 50 mM Tris/MES buffer.
- Acetaminophen was dissolved to a final concentration of 1 mM in banana pulp supernatant at each pH. The supernatants were then incubated at 37°C for 0–60 min. The residual acetaminophen was then determined. The experiment was conducted three times.

d) Differences in acetaminophen loss rate among various banana types

- Commercially obtained bananas grown in the Philippines, Taiwan, Peru, Ecuador, Colombia, and Mexico (34 varieties) were homogenized and the resulting pulp was centrifuged at 16,000 g. A commercial banana beverage was also centrifuged at 16,000 g. The resulting supernatants were used as stock solutions.
- Acetaminophen was dissolved to a final concentration of 1 mM in each 50% banana pulp supernatant. These supernatants were then incubated at 37°C for 30 min. The residual acetaminophen was then measured. The experiment was conducted three times.

### Detection of reaction products

- Identification of 3-hydroxyacetaminophen, an assumed reaction product derived from acetaminophen, in solutions containing banana extract, was attempted using LC/ESI/MS as described above [8].
- Acetaminophen was dissolved to a final concentration of 1 mM in 50% supernatants prepared from pulp homogenate of bananas grown in the Philippines. The supernatants were then incubated at 37°C for 20 min in the presence of ascorbic acid (0.01–100mM).
- Nine times the amount of acetonitrile was then added to the solution and the reaction products were determined using LC/ESI/MS (m/z scan mode, positive and negative).

### Statistical analysis

- The mean (± standard deviation) was calculated for all continuous variables. The Student’s *t*-test was used for single comparison tests, and the Dunnett’s test [7] was used for multiple comparison tests. *P* < 0.05 was considered statistically significant. All statistical analyses were performed using JMP®Pro14 (SAS Institute Inc., NC, USA).

## Results

### Characterization of incompatibility

a) Incompatibility observed after enzyme deactivation in banana extract

**Fig 2:**
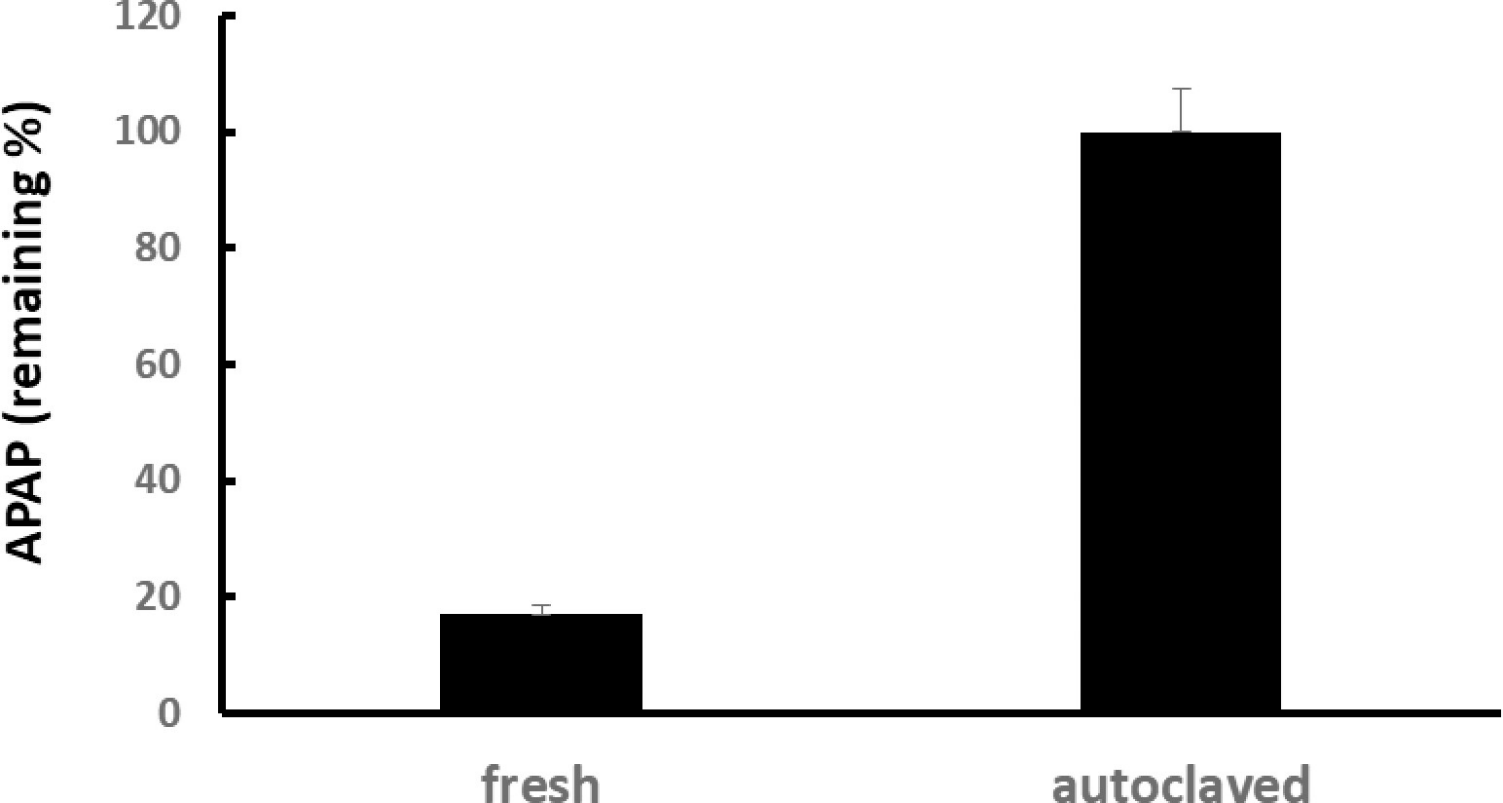
Effect of heating banana extract on acetaminophen loss. Difference in acetaminophen loss between fresh and autoclaved banana juice. Values are shown as percentage of acetaminophen remaining after addition to fresh or autoclaved juice (mean of triplicates ± 1 standard deviation [SD]). APAP: acetaminophen.

Acetaminophen residual concentrations in fresh and autoclaved banana pulp supernatants are shown in Fig 2. More than 80% of acetaminophen loss was observed in fresh banana pulp supernatant, whereas the amount of acetaminophen did not change in autoclaved banana pulp supernatant. The difference between residual concentrations was statistically significant (*P* < 0.0001).

b) Effect of temperature on acetaminophen loss in banana extract

**Fig 3:**
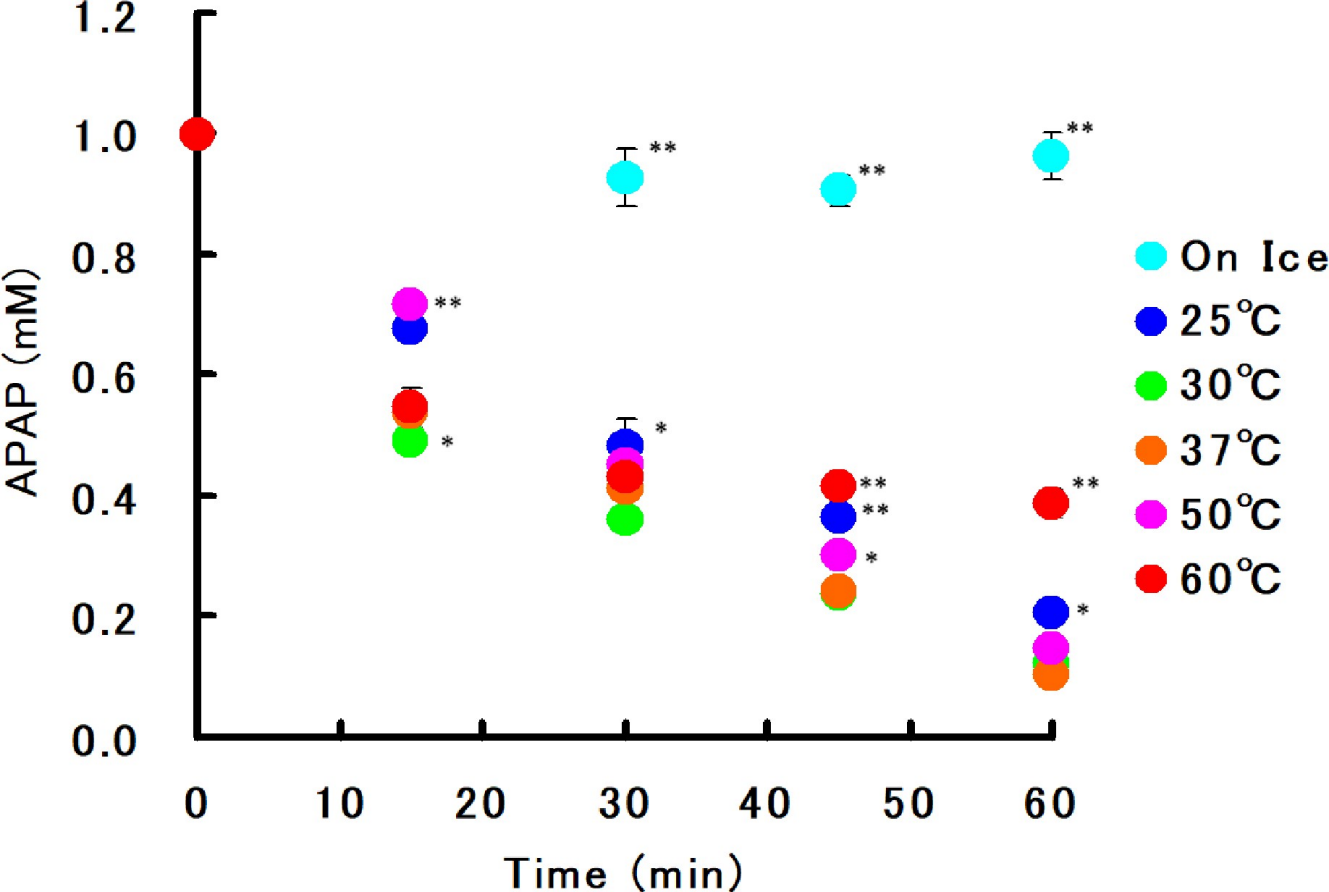
Effect of incubation temperature on acetaminophen loss in banana extract. Temporal changes in acetaminophen concentration (1 mM initial concentration in 50% banana supernatant) at various incubation temperatures were monitored (Mean of triplicates ± 1 SD). Significant differences between 37°C and other values are shown as * (*P* < 0.05) and ** (*P* < 0.001). APAP: acetaminophen.

Temporal changes in residual acetaminophen in banana pulp supernatants under various temperature conditions are shown in Fig 3. Incompatibility was not observed under ice-cold conditions. In addition, optimum reaction temperatures appeared to be 30°C–37°C.

c) Effect of pH on acetaminophen loss in banana extract

**Fig 4:**
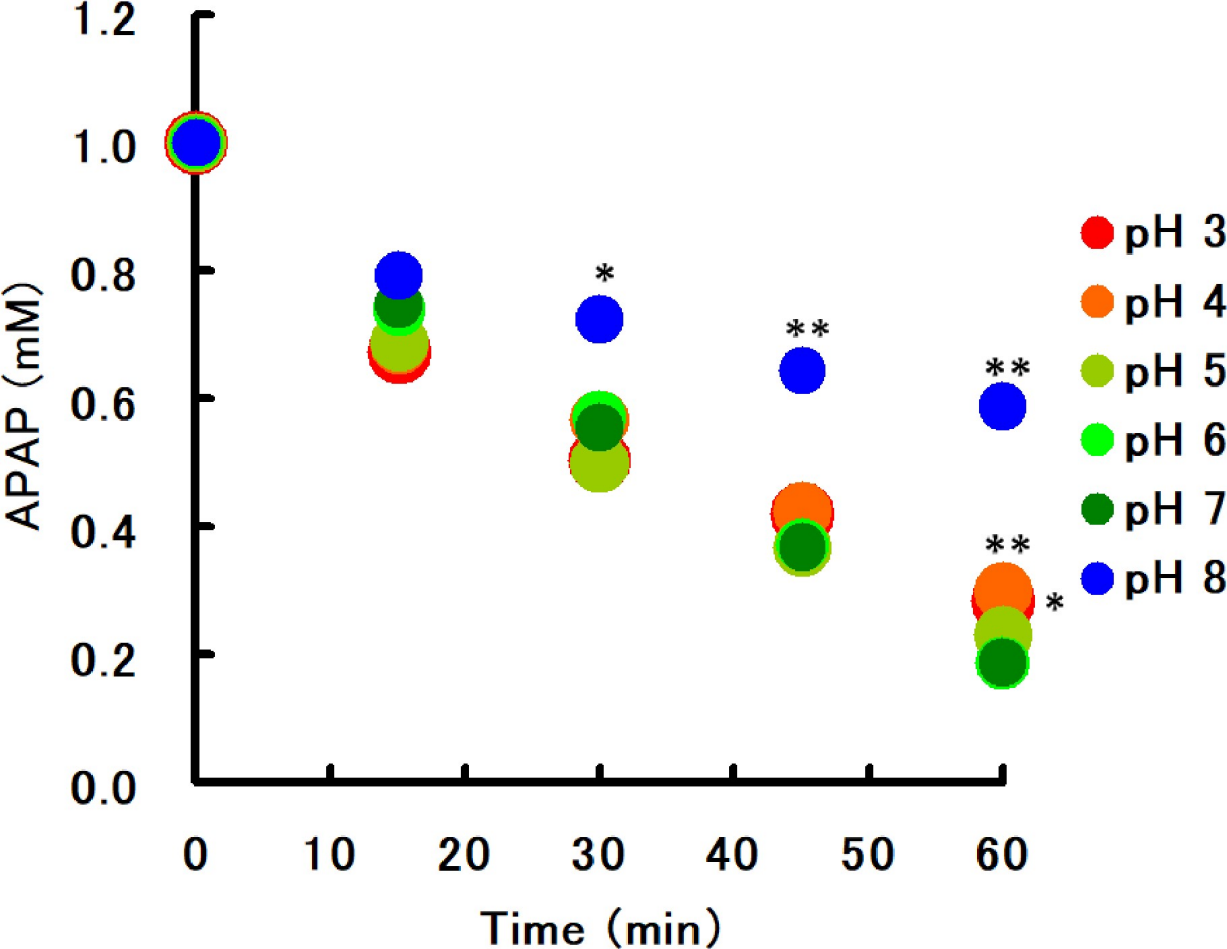
Effect of pH on acetaminophen loss in banana extract. Temporal changes in acetaminophen concentrations (1 mM initial concentration in 50% banana supernatant) at various pH values were monitored (mean of triplicates ± 1 SD). Significant differences between pH 7.0 and other values are shown as * (*P* < 0.05) and ** (*P* < 0.001). APAP: acetaminophen.

Temporal changes in residual acetaminophen in banana pulp supernatants under various pH conditions are shown in Fig 4. Reactions reducing acetaminophen potency readily proceeded in a wide pH range, from neutral to acidic conditions.

d) Differences in acetaminophen loss rate among various banana types

**Fig 5:**
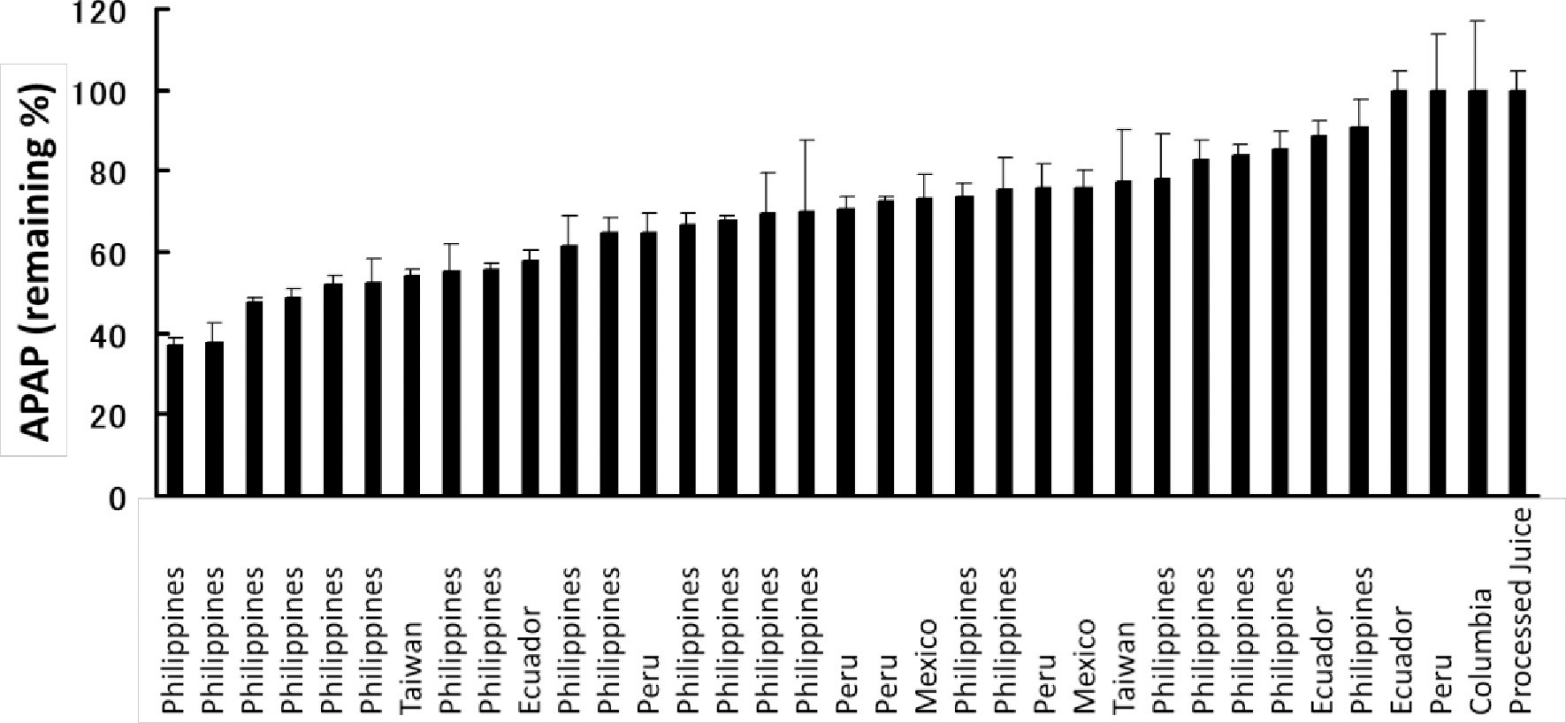
Effect of banana type. Acetaminophen concentrations (1 mM initial concentration in 50% banana supernatant) remaining after incubation at 30°C for 30 min were determined in various banana types (mean of triplicates ± 1 SD). Countries of origin of the bananas are indicated. APAP: acetaminophen.

Residual acetaminophen in banana pulp supernatants from various bananas types is shown in Fig 5. We found substantial incompatibility in all types, but the degree depended on the banana type. We observed a relatively high interaction activity in bananas grown in the Philippines. The average residual acetaminophen concentration after incubation with bananas from Asian countries such as the Philippines and Taiwan (64.8% ± 15.3%) was significantly lower than that from American countries such as Ecuador, Peru, Mexico, and Columbia (80.0% ± 15.3%) (*P* = 0.0096).

### Reaction detection

**Fig 6:**
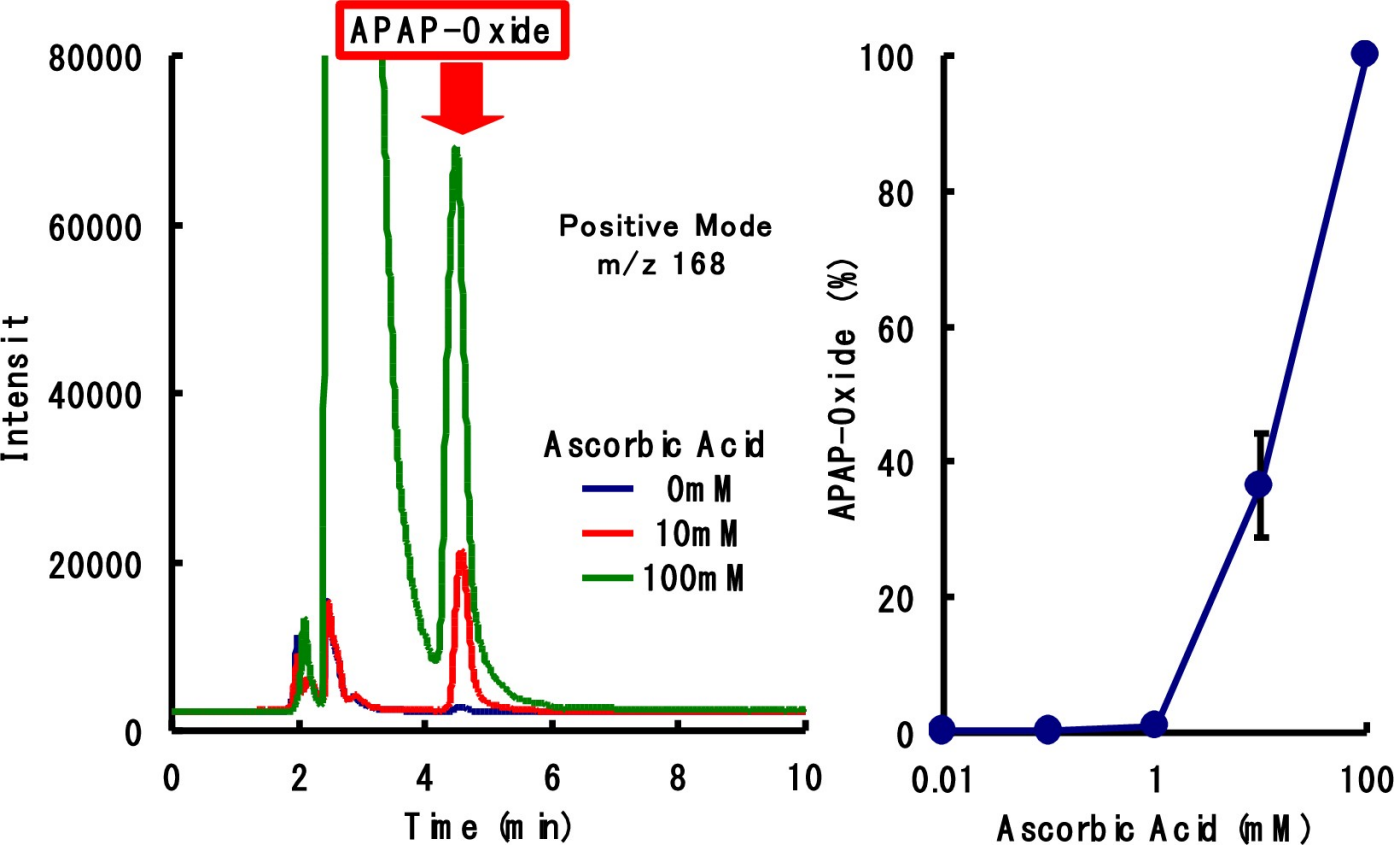
Effect of ascorbic acid on 3-hydroxyacetaminophen formation in banana–acetaminophen incompatibility. 3-hydroxyacetaminophen production in the presence of various ascorbic acid concentrations were measured by the LC/ESI/MS method. APAP: acetaminophen.

**Fig 7:**
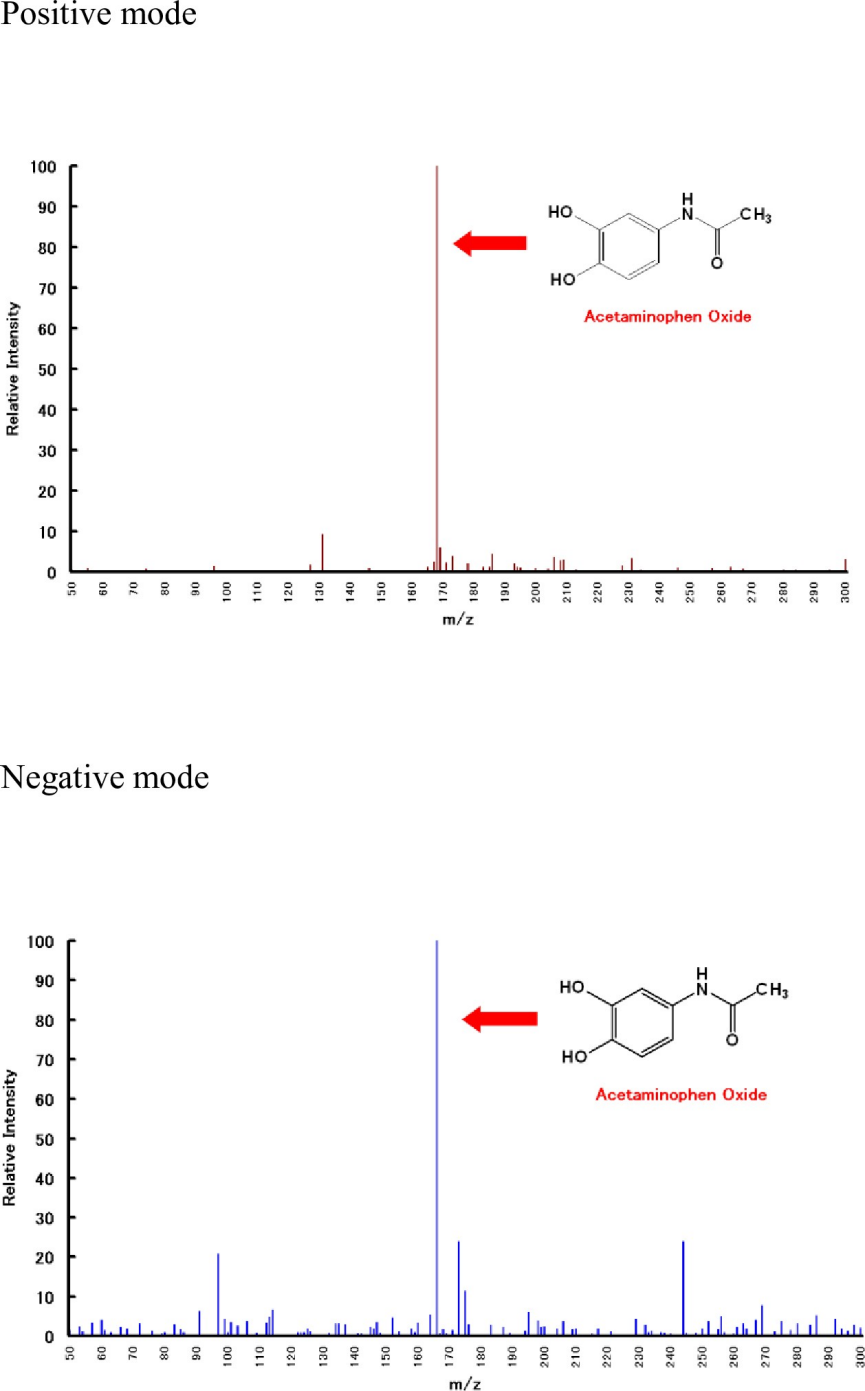
Detection of 3-hydroxyacetaminophen ion produced in banana–acetaminophen incompatibility. Both positive and negative modes of LC/ESI/MS were used. Further details are provided in the Methods of this paper.

LC/ESI/MS chromatogram analysis revealed that additions of increasing amounts of ascorbic acid resulted in a single peak retention time of 4.3 min (Fig 6). We observed major molecular ions at m/z:168 in the positive mode and m/z:166 in the negative mode after analysis of this peak component in m/z ratio scan mode (Fig 7). These results were consistent with those obtained from 3-hydroxyacetaminophen products.

## Discussion

In this study, we examined various factors in an effort to explore the mechanisms underlying banana–acetaminophen incompatibility and the effect on acetaminophen intake. Results from the present study confirm that the potency of acetaminophen rapidly diminishes when incorporated in banana extract (Figs 2 and 3). Notably, this is the first study showing that the decomposition of acetaminophen can be attributed to metabolic enzymes present in food.

A change in incompatibility was observed in banana supernatant after autoclaving, a treatment aimed to achieve enzyme deactivation. Our results indicate that the acetaminophen inactivation activity of the bananas disappeared after autoclaving (Fig 2). Furthermore, the reaction was temperature-dependent, with optimal temperatures being 30–37°C. However, the reaction was absent under ice-cold temperatures (Fig 3). In addition, our results indicate that the reaction is pH-dependent (Fig 4).

**Fig 8:**
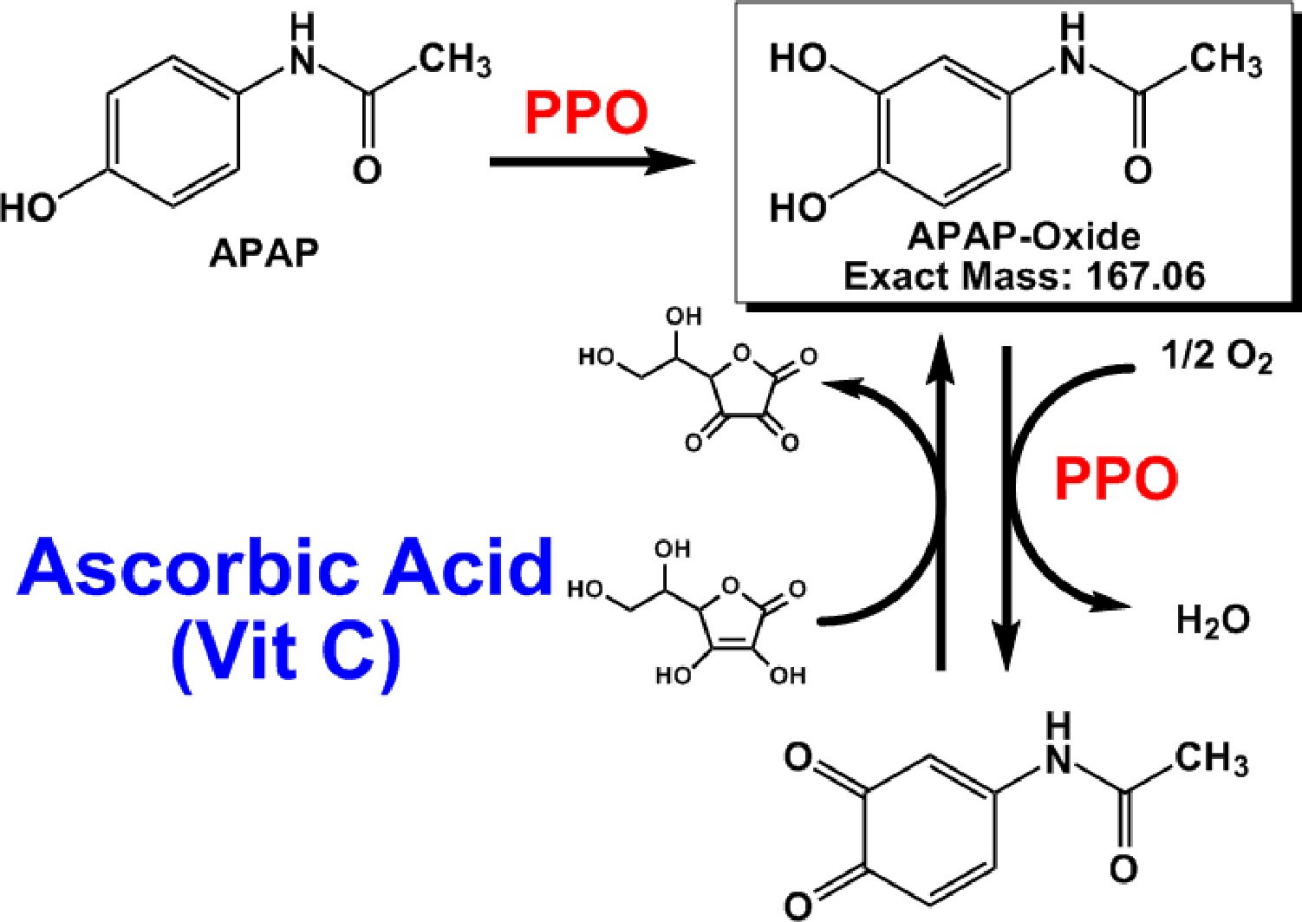
Estimated oxidation reaction pathway of acetaminophen. APAP: acetaminophen.

Thus, our observations suggest that the incompatibility is due to an enzymatic reaction. Polyphenol oxidase found in bananas causes loss of levodopa potency by impacting levodopa bioavailability [2]. Previously, we reported that similar to levodopa, the potency of α-methyldopa, a catecholic oral drug, was rapidly diminished by enzymes present in bananas [3]. Meanwhile, mushroom-derived tyrosinase was determined to catalyze the catecholation reaction by the oxidation of acetaminophen [8, 9]. In our study, loss of acetaminophen potency in the presence of bananas may be due to the abundance of polyphenol oxidase, which normally catalyzes the conversion of tyrosine (Fig 1). Thus, we aimed to detect 3-hydroxyacetaminofen, a metabolite derived from acetaminophen, through studying the tyrosinase activity of polyphenol oxidase. Indeed, ions derived from the metabolite were confirmed on the LC/ESI/MS spectrum (Fig 7). The oxidation reaction catalyzed by 3-hydroxyacetaminofen tyrosinase activity is reported to be reversibly inhibited by ascorbic acid [8]. Conversely, to prove that the tyrosinase in bananas causes the interaction, we must show that PPO from bananas converts acetaminophen into APAP oxide. Further research involving enzyme purification and activity measurement is expected to support this mechanism. We propose that a similar mechanism (Fig 8) occurs in the interaction observed here. Since ascorbic acid inhibits the polymerization reaction, the concentration of 3-hydroxyacetaminophen as a reaction intermediate may presumably increase. In fact, our study showed that the generation of the reaction intermediate was dependent on the amount of ascorbic acid added (Fig 6). This result strongly suggests the involvement of polyphenol oxidase in the incompatibility.

We found extremely diverse enzyme activities depending on banana type after studying acetaminophen loss in a variety of commercially available bananas (Fig 5). This diverse activity appears to be due to the polyphenol oxidase activity of each banana type. We observed high polyphenol oxidase activities in bananas grown in the Philippines, whereas low activities were noted in bananas grown in South America. Since most bananas imported to Japan originate from the Philippines, our findings suggest that banana intake may affect the medicinal properties of cold medicine in Japan.

Importantly, the incompatibility observed in this study is a potency reduction reaction that occurs *in vitro*. However, our results show that the reaction readily proceeds even at an acidic pH (Fig 4), and optimum temperature for the interaction are near body temperature (Fig 3). Thus, it is possible that after banana intake, the reaction with acetaminophen medication may proceed in the stomach as described here. We attempted to confirm this interaction *in vivo* in a preliminary pharmacokinetic experiment using rats by concomitant administration of a banana supernatant and a commercially available OTC acetaminophen aqueous solution; our results showed a significant decrease in area under the curve of acetaminophen (data not shown). Ogo et al. [2] reported similar findings and showed that banana extract diminished the biological utilization of levodopa in animal experiments. Thus, this study and our observations suggest that banana–acetaminophen incompatibility may occur in the gastrointestinal tract.

Notably, scenarios wherein banana extract and acetaminophen are mixed may be uncommon. However, bananas are often used in baby food. Therefore, interaction may have an impact on the efficacy of cold medications, especially for pediatric applications. The discovery of such major food and drug interactions is presumably delayed since communication between affected children and parents is insufficient.

## Conclusion

In this study, bananas were found to diminish acetaminophen potency, and the incompatibility appeared to be due to an enzymatic reaction catalyzed by polyphenol oxidase contained in banana pulp. Our study also revealed that the rate of decrease in acetaminophen potency widely varies with banana type. Additionally, the interaction can occur with commercial OTC cold medications and may progress in the gastrointestinal tract after banana intake. Importantly, further studies are required since clinical findings on the effects of banana intake on acetaminophen pharmacokinetic behavior in humans are currently lacking.
